# Cognitive Reactivity to Success and Failure Relate Uniquely to Manic and Depression Tendencies and Combine in Bipolar Tendencies

**DOI:** 10.1155/2012/753946

**Published:** 2011-12-07

**Authors:** Filip Raes, Ine Ghesquière, Dinska Van Gucht

**Affiliations:** ^1^Department of Psychology, University of Leuven, 3000 Leuven, Belgium; ^2^The Research Foundation Flanders (FWO), 1000 Brussels, Belgium

## Abstract

The present study examined simultaneously the relations between cognitive reactivity to success and failure, on the one hand, and depression, manic, and bipolar tendencies, on the other hand. Participants (161 students) completed measures of success and failure reactivity, current manic and depressive symptoms, and tendencies towards depression, mania, and bipolarity. Results showed that respondents with a greater tendency towards depression evidenced greater (negative) reactivity to failure, whereas those with a greater tendency toward mania evidenced greater (positive) reactivity to success. Depression vulnerability was unrelated to success reactivity, and manic vulnerability was unrelated to failure reactivity. Tendencies toward bipolarity correlated significantly with *both* failure and success reactivity in a negative and positive manner, respectively. These findings add to the growing body of literature, suggesting that different features or cognitive tendencies are related to depression vulnerability versus manic vulnerability and imply that these “mirrored” cognitive features both form part of vulnerability to bipolar disorder.

## 1. Introduction

A key feature of depression vulnerability is increased cognitive reactivity to negative events or negative mood [1, 2]. It refers to the degree to which a mild dysphoric state reactivates negative cognitions. It is believed that such increased cognitive reactivity exacerbates negative emotion and, that way, precipitates depressive episodes [3, 4]. Parallel to such a pattern of increased *negative* reactivity to negative events, more recent findings from a largely separate literature suggest that patients with bipolar disorder experience greater reactivity to *positive* events (see [5] for a review), which, in turn, might boost positive emotion and, that way, increase manic symptoms over time [6]. 

Whereas the association between negative reactivity and depression (vulnerability) has been extensively studied and is well established, research on the relationship between positive reactivity and mania/bipolarity is clearly lagging behind. Also, negative and positive reactivity have been studied in largely separate literatures, focusing either on (unipolar) depression or on bipolar depression and mania, thereby limiting potential integration and crucial linking of important patterns of findings. Given that these two “mirrored” forms of cognitive reactivity have been put forward as potentially relevant in explaining vulnerability to depression and mania, much more insight could be gained from research that simultaneously focuses on positive and negative reactivity in relation to both depression and mania. Although sorely needed, such studies are rare. Eisner et al. [7] have recently started to examine positive and negative reactivity (focusing on success and failure reactivity) in relation to both mania and depression. They showed that increased reactivity to failure was associated to depression vulnerability, whereas increased reactivity to success was uniquely related to mania vulnerability. Whereas Eisner et al. [7] still investigated success and failure reactivity in relation to mania and depression separately (one study focusing on failure reactivity and the other on success reactivity), Carver and Johnson [8] recently studied the associations in one and the same sample in a single study, replicating the findings of Eisner et al. [7]. 

Eisner et al. [7] rightly cautioned that their findings should be considered preliminary and, thus, that replication is needed by independent researchers preferentially using different measures for manic/depression tendencies and success/failure reactivity. This is precisely what the present study set out to do. In a sample of Belgian high school students, we administered a new measure that we constructed to assess failure and success reactivity (SFRS, see below) and a measure to assess depression and manic tendencies which was different to the ones used previously [7, 8]. Consistent with Eisner et al. [7], we hypothesized that failure, but not success reactivity, would be related to depression tendencies, whereas manic tendencies would be related to success but not to failure reactivity. 

Besides extending the previous findings using different measures, the current study attempted to take the previous work a step further in yet another important way. The mania/depression measure used in the present study includes, besides items assessing propensity to either mania or depression symptoms, also the so-called biphasic items that assess fluctuation between depressive and hypomanic states (i.e., propensity towards bipolarity). The latter allowed us to test, for the first time, whether failure and success reactivity, which are supposed to *uniquely* relate to depression, and manic tendencies, respectively, are *both* associated to tendencies to bipolarity. Given that depression (vulnerability) is characterized by negative reactivity and mania (vulnerability) by positive reactivity, those who are characterized by both tendencies toward depression and mania (and thus bipolarity) are expected to exhibit positive as well as negative reactivity. Thus, besides our hypotheses that failure, but not success reactivity, would be related to depression tendencies and that manic tendencies would be related to success, but not to failure reactivity, we additionally hypothesized that tendencies toward bipolarity would be related to *both* forms of reactivity. Finally, we hypothesized that these associations would remain even after controlling for current symptoms of depression and mania.

## 2. Method

### 2.1. Participants and Procedure

Participants were 161 Belgian Dutch-speaking students from the last two years of secondary school (105 women, 56 men). The average age was 16.68 years (SD = 0.67; range: 16–19). All respondents participated without compensation. Following informed consent, participants completed all measures (see below) at home.

### 2.2. Measures

#### 2.2.1. General Behavior Inventory (GBI)

The GBI [9] assesses unipolar and bipolar affective conditions on trait or lifetime basis. It contains 73 items, which comprise three subscales. A first subscale of 45 items measures symptomatic behaviours associated with depression (e.g., “Have there been times of several days or more when you really got down on yourself?”). A second subscale of 21 items measures symptomatic behaviours associated with (hypo)mania (e.g., “Have there been periods of several days or more when your friends or family told you that you seemed unusually happy or high-clearly different from your usual self or from a typical good mood?”). A third subscale of 7 biphasic items measures fluctuation between both depressive and hypomanic behaviours (e.g., “Have you had periods lasting several days or more when you felt depressed or irritable, and then other periods of several days or more when you felt extremely high, elated, and overflowing with energy?”). Items are rated on a 4-point scale, *never or hardly ever *(1),* sometimes* (2), *often* (3), and *very often *(4). The four alternatives are scored 0, 0, 1, and 1 [9]. Adequate psychometrics are reported for the original English GBI [9] as well as for the Dutch version [10]. Cronbach's alphas in the present study were  .92,  .82, and  .72 for the depression, (hypo)mania, and bipolar/biphasic scale, respectively. ^1^ As the GBI includes items focused on a lifetime history of depression, (hypo)manic, and biphasic/bipolar symptoms, scores on each of these scales were, following Carver and Johnson [8], conceptualized as tendencies towards these affective conditions, or risks or vulnerabilities for these conditions. 

#### 2.2.2. Depression Anxiety Stress Scale (DASS)

The DASS is a 21-item self-report instrument to assess current (past week) depression, anxiety, and stress symptomatology [11]. Each of the three subscales consists of seven items, all scored on 0–3 scale. Good psychometric properties are reported [11]. Only the Depression subscale (DASS-D) of the Dutch version by de Beurs et al. [12] was used. Cronbach's alpha in the present sample was  .82.

#### 2.2.3. Altman Self-Rating Mania Scale (ASRM)

The ASRM [13] assesses current (past week) manic symptoms using five items (increased cheerfulness, inflated self-confidence, talkativeness, reduced need for sleep, and excessive behavioral activity). Each item consists of a group of five statements with increasingly severe descriptions (0–5 scale). The ASRM has good psychometric properties and correlates strongly with clinician-administered ratings [13]. The English ASRM was translated into Dutch by F. Raes and D. V. Gucht (FR and DVG). Next, the Dutch ASRM was translated back into English by Professor Dr. Kristin Blanpain, a native Dutch speaker with a Ph.D. degree in English Literature and extensive expertise in translating and revising academic documents (including backtranslation of questionnaires). Finally, the backtranslation was evaluated and approved by Dr. Altman, the main author of the original English version. Cronbach's alpha in the present sample was  .76, comparable to internal consistency values reported in the literature for the English version (e.g.,  .70; [14]).

#### 2.2.4. Success and Failure Reactivity Scales (SFRS)

Respondents are asked to imagine that they feel neither particularly sad nor particularly cheerful and that they *fail* at something which is important to them. Then, they are instructed to keep this situation in mind when indicating how they would typically feel/think about themselves after such a *failure experience* on two −10 to +10 rating scales:* I feel much less self assured than before* (−10) over *I feel as self assured as before* (0) to *I feel much more self assured than before *(+10) and *I think I'm not good at anything at all *(−10), over *I still think the same about myself *(0), to *I think I can achieve everything* (+10). Success reactivity is assessed using the same two items. Now, respondents are asked to imagine that they feel neither particularly sad nor particularly cheerful and that they *succeed* at something which is important to them. They are then instructed to keep this situation in mind when indicating how they would typically feel/think about themselves after such a *success experience* using the same two −10 to +10 rating scales with identical anchor points. A total failure reactivity score is derived averaging both failure item scores (Cronbach's alpha =  .76; *r* = .61). Likewise, a total success reactivity score is obtained averaging both success item scores (Cronbach's alpha =  .84; *r* = .73). 

## 3. Results

Mean scores, standard deviations and ranges for all variables included were as follows: GBI depression (*n* = 151; M = 6.22; SD = 6.92; range = 0–32); GBI (hypo)mania (*n* = 151; M = 3.30; SD = 3.45; range = 0–17); GBI biphasic (*n* = 156; M = 1.50; SD = 1.73; range = 0–7); DASS-D (*n* = 161; M = 3.60; SD = 3.61; range = 0–18); ASRM (*n* = 161; M = 4.50; SD = 3.46; range = 0–19); failure reactivity (*n* = 161; M = −3.55; SD = 2.66; range = −10–5); success reactivity (*n* = 161; M = 4.48; SD = 2.43; range = −2–10).

As predicted, tendencies toward depression (GBI depression scores) correlated significantly negatively with failure reactivity scores but were unrelated to success reactivity scores (see Table 1). Second, tendencies toward mania (GBI (hypo)mania), on the other hand, were unrelated to failure reactivity, but correlated significantly positively with success reactivity. Third, tendencies toward bipolarity (GBI biphasic scores) correlated significantly with both failure and success reactivity in a negative and positive manner, respectively. Finally, these associations remained after controlling for current symptoms of depression (DASS-D scores) and mania (ASRM scores) (also see Table 1), indicating that the observed associations are not attributable to current mood symptoms.

## 4. Discussion

The present study examined simultaneously the relations between success and failure reactivity, on the one hand, and depression, manic, and bipolar tendencies, on the other hand. People with a greater tendency toward depression evidenced greater (negative) reactivity to failure, whereas people with a greater tendency toward mania evidenced greater (positive) reactivity to success. Depression vulnerability was unrelated to success reactivity, and manic vulnerability was unrelated to failure reactivity. Thus, success and failure reactivity relate uniquely to manic versus depression tendencies, which is consistent with earlier findings by Eisner et al. [7] and Carver and Johnson [8]. The present results further extend these prior findings to a different sample using different measures to assess depression and manic tendencies and success and failure reactivity. Furthermore, unlike Eisner et al. [7], we established these unique relationships for success and failure reactivity in one and the same sample [8]. Together with the study of Carver and Johnson [8], the present study represents one of the rare comprehensive studies in which positive and negative cognitive tendencies are jointly studied in relation to both mania and depression, which adds to its importance. Of most importance, the present study was the first to examine and establish the *combined *existence of failure and success reactivity in bipolar tendencies, separate from depression and manic tendencies: tendencies toward bipolarity correlated significantly with *both* failure and success reactivity in a negative and positive manner, respectively.

These findings suggest that patients with bipolar disorder, or with a propensity towards this diagnosis, may have an increased reactivity to both success and failure reactivity, of which only the latter is shared by patients suffering from unipolar depression or people with a propensity towards unipolar depression. Just as that increased reactivity to failure (and to other negatively valenced events in general) can precipitate and exacerbate depressive symptoms, increased reactivity to success (and other positive events more generally) may precipitate (hypo)manic episodes. Thus, people who score high on bipolar tendency are characterized by both increased negative and positive reactivity to failure and success, respectively, which may underlie their experiencing of both depressed and (hypo)manic episodes.

The current study has two potential limitations that are noteworthy. The first is that we only relied on self-report measures to assess mania/depression and success/failure reactivity. Secondly, the present results were obtained in a student population, limiting the generalizability to, for example, more clinical populations. As such, future research should test the replicability of these findings using clinician-administered instruments (mania/depression) and behavioral laboratory paradigms (e.g., experimental induction of failure and success experiences; [15, 16]) in both clinical and control/community samples.

In summary, the current study examined and established, for the first time to our knowledge, the *combined *existence of failure and success reactivity in bipolar tendencies, two contrasting forms of reactivity that each uniquely relates to depression and manic tendencies, respectively. The present findings, then, add to the growing body of literature suggesting that different features or cognitive tendencies are related to depression vulnerability versus manic vulnerability [7, 8] and suggest that these “mirrored” cognitive features both form part of vulnerability to bipolar disorder. 

## Figures and Tables

**Table 1:** Correlations and partial correlations (current manic and depression symptoms partialled) between depression (*n* = 151), (hypo)mania (*n* = 151), and biphasic (*n* = 156) GBI-scores and failure and success reactivity.

	Failure reactivity^a^	Success reactivity^a^
GBI depression	−.24** (−.20*)	.09
GBI (hypo)mania	−.06	.33*** (.32***)
GBI biphasic	−.26** (−.23**)	.16* (.26**)

GBI: general behavior inventory; values between brackets are partial correlations between variables controlled for current manic (ASRM) and depression (DASS-D) symptoms; n varies because of missing data.

^a^Higher scores on the reactivity scales reflect higher levels of self-assurance/self-confidence; lower scores reflect lower levels of self-assurance/self-confidence;

*P < .05.  **P < .01.  ***P < .001.

## Endnotes

1. We also developed a shortened 28-item version of the 73-item GBI which could be of use in time and cost intensive survey research. Similar to the full GBI, the GBI short form (GBI-SF) contains three sets of items: 14 depression items (items 3, 14, 16, 23, 32, 34, 36, 55, 56, 62, 63, 68, 71, and 73), 7 hypomanic items (items 4, 8, 11, 30, 38, 54, and 61), and 7 biphasic items (same items as in the original GBI: 2, 19, 24, 35, 40, 48, and 53). Cronbach's alphas for the shortened depression and hypomania scales are  .90 and  .72, respectively. Correlations between the depression and hypomania subscales for the long and short form were  .94 and  .92, respectively. Also, the pattern of correlations with, for example, current depression and mania symptoms did not significantly change when using the shortened subscales.
